# Biochemical and Genetic Analysis of 4-Hydroxypyridine Catabolism in *Arthrobacter* sp. Strain IN13

**DOI:** 10.3390/microorganisms8060888

**Published:** 2020-06-12

**Authors:** Justas Vaitekūnas, Renata Gasparavičiūtė, Jonita Stankevičiūtė, Gintaras Urbelis, Rolandas Meškys

**Affiliations:** 1 Department of Molecular Microbiology and Biotechnology, Institute of Biochemistry, Life Sciences Center, Vilnius University, Saulėtekio al. 7, LT-10257 Vilnius, Lithuania; renata.gasparaviciute@bchi.vu.lt (R.G.); jonita.stankeviciute@bchi.vu.lt (J.S.); rolandas.meskys@bchi.vu.lt (R.M.); 2 Department of Organic Chemistry, Center for Physical Sciences and Technology, Akademijos 7, LT-08412 Vilnius, Lithuania; gintaras.urbelis@ftmc.lt

**Keywords:** 4-hydroxypyridine, 3,4-dihydroxypyridine, 4-hydroxypyridine 3-monooxygenase, 3,4-dihydroxypyridine dioxygenase, extradiol dioxygenase, amidohydrolase, biodegradation, *Arthrobacter*

## Abstract

*N*-Heterocyclic compounds are widely spread in the biosphere, being constituents of alkaloids, cofactors, allelochemicals, and artificial substances. However, the fate of such compounds including a catabolism of hydroxylated pyridines is not yet fully understood. *Arthrobacter* sp. IN13 is capable of using 4-hydroxypyridine as a sole source of carbon and energy. Three substrate-inducible proteins were detected by comparing protein expression profiles, and peptide mass fingerprinting was performed using MS/MS. After partial sequencing of the genome, we were able to locate genes encoding 4-hydroxypyridine-inducible proteins and identify the *kpi* gene cluster consisting of 16 open reading frames. The recombinant expression of genes from this locus in *Escherichia coli* and *Rhodococcus erytropolis* SQ1 allowed an elucidation of the biochemical functions of the proteins. We report that in *Arthrobacter* sp. IN13, the initial hydroxylation of 4-hydroxypyridine is catalyzed by a flavin-dependent monooxygenase (KpiA). A product of the monooxygenase reaction is identified as 3,4-dihydroxypyridine, and a subsequent oxidative opening of the ring is performed by a hypothetical amidohydrolase (KpiC). The 3-(*N*-formyl)-formiminopyruvate formed in this reaction is further converted by KpiB hydrolase to 3-formylpyruvate. Thus, the degradation of 4-hydroxypyridine in *Arthrobacter* sp. IN13 was analyzed at genetic and biochemical levels, elucidating this catabolic pathway.

## 1. Introduction

The derivatives of pyridine are ubiquitous in nature—from universally used cofactors such as nicotinamide adenine dinucleotide (NAD) and pyridoxal phosphate (PLP) to specific plant alkaloids, including nicotine, actinidine, mimosine, etc. Furthermore, synthetic pyridine compounds are widely used as dyes, explosives, pharmaceuticals, and pesticides [1].

The prevalence of 4-hydroxypyridine (4HP) in nature has not been substantially studied. Nevertheless, this compound has been identified in *Arabidopsis thaliana* [2] and *Arabidopsis lyrata* ssp. *petraea* [3] extracts. It has also been detected as an unspecific detoxification product of pyridine in laboratory animals [4]. Among other compounds, 4HP has been selected as a biomarker of increasing ocean acidification [5]. A methylated derivative of 4HP has been isolated from the culture filtrate of the fungus *Physisporinus sanguinolentus* [6]. In addition, the derivatives of 4HP enter the environment from several other sources: the synthetic herbicide pyrichlor (2,3,5-trichloro-4-hydroxypyridine), the radiocontrast agent diodone (2-(3,5-diiodo-4-oxopyridin-1-yl)acetate), a natural allelochemical mimosine [7], natural antibiotics caerulomycin [8] and piericidin [9], and an atypical NAD metabolite (4-pyridone-3-carboxamide-1-β-D-ribonucleoside triphosphate) [10]. These compounds do not accumulate to a large extent in nature, which implies that eventually they are degraded by abiotic factors and the microbiome. Therefore, bioremediation of persistent pollutants is of considerable practical interest. It is well known that many soil bacteria can degrade various *N*-heterocycles, such as pyridine, nicotine, and mimosine [1]. Apart from the benefits of mineralization of the toxic xenobiotics, these microbial activities could be implemented in modern biocatalysis. Regiospecific hydroxylation of various aromatic compounds using environmentally friendly catalysts is especially desirable in organic chemistry [11].

It has been reported previously that *Agrobacterium* sp. (NCIB 10413) can use 4HP as a sole carbon and energy source [12]. The investigation of 4HP metabolism in this bacterium using cell-free extracts has revealed that the first reaction is catalyzed by a flavin adenine dinucleotide (FAD)-dependent monooxygenase, and the product of this reaction has been identified as 3,4-dihydroxypyridine (34DHP) [13]. The subsequent catabolism involves a ring-opening dioxygenase and a hydrolase. The end products of 4HP degradation in *Agrobacterium* sp. have been determined to be NH_3_, formate, and pyruvate [14]. The intermediate product of mimosine degradation in *Rhizobium* sp. strain TAL1145 is also 34DHP [15], which is further metabolized by the PydA dioxygenase and PydB hydrolase [16]. An examination of 4-aminopyridine-degrading enrichment culture from a soil sample has indicated that 34DHP and formate are the probable metabolites [17]. However, the precise genes or proteins responsible for 4HP biodegradation have not been reported yet.

In this study, we report the characterization of the 4HP catabolic pathway in *Arthrobacter* sp. IN13, which has been isolated on the basis of its ability to utilize 4HP as a carbon source for growth [18]. A gene cluster (*kpi*) encoding the proteins required for 4HP biodegradation was identified in this bacterium, and the resulting proteins were characterized. The metabolic pathway is proposed on the basis of the identification of the intermediate metabolites. We demonstrate that the flavin-dependent monooxygenase KpiA is responsible for the initial step of 4HP biodegradation. We also describe an enzymatic ring-opening reaction of 34DHP that is catalyzed by the hypothetical amidohydrolase KpiC.

## 2. Materials and Methods

### 2.1. Bacterial Strains, Plasmids, Primers, and Standard Techniques

The 4HP-degrading bacterium *Arthrobacter* sp. IN13 was isolated from a soil sample [18]. *Rhodococcus erythropolis* strain SQ1 was chosen as the host strain for the expression of the recombinant genes for bioconversion experiments. *Escherichia coli* strain DH5α was used for cloning experiments. The recombinant proteins were overexpressed in *E*. *coli* strain BL21 (DE3). The bacterial strains, plasmids, and primers used in this study are listed in Table S1 in the Supplementary Materials section. Standard molecular biology techniques were performed as previously described [19].

### 2.2. Bacterial Growth Medium and Conditions

Mineral medium (MM) (g L^−1^): NaCl 5.0, NH_4_H_2_PO_4_ 1.0, K_2_HPO_4_ 1.0, MgSO_4_ × 7H_2_O 0.4, pH 7.2; 100× salt solution (g L^−1^): CaCl_2_ × 2H_2_O 2.0, MnSO_4_ × 4H_2_O 1.0, FeSO_4_ × 7H_2_O 0.5—all components were dissolved in 0.1 N HCl and added into MM after sterilization. *R*. *erythropolis* SQ1 was grown at 30 °C with aeration, and *E*. *coli* strains were grown at 37 °C with aeration. *Arthrobacter* sp. IN13 was cultivated in minimal medium supplemented with either 4HP (0.2%) or succinate (0.1%). *E*. *coli* strains transformed with the recombinant plasmids were grown in nutrient broth medium supplemented with either 50 μg mL^−1^ ampicillin or 40 μg mL^−1^ kanamycin, as required. *R*. *erythropolis* SQ1 transformed with recombinant plasmids was grown in the presence of 30 μg mL^−1^ chloramphenicol. *R*. *erythropolis* SQ1 and *E*. *coli* strains were transformed with plasmid DNA by electroporation.

### 2.3. Analysis of the Protein Expression Profile Induced by 4HP

*Arthrobacter* sp. IN13 was cultivated in minimal medium supplemented with either 4HP or succinate; cells were collected by centrifugation and suspended in 50 mM potassium phosphate, pH 7.2. Cells were disrupted by sonication. Cell debris was removed by centrifugation at 16,000× *g* for 10 min. Proteins were separated on 14% SDS-PAGE gel and visualized by Coomassie blue staining. The bands corresponding to the induced proteins were excised and subjected to de novo sequencing based on matrix-assisted laser desorption ionization–time of flight (MALDI-TOF)/TOF mass spectrometry (MS) and subsequent computational analysis at the Proteomics Centre of the Institute of Biochemistry, Life Science Center, Vilnius University (Vilnius, Lithuania), as described [20].

### 2.4. Gene and Protein Sequence Analysis

*Arthrobacter* sp. IN13 DNA was sequenced using an Illumina platform, and contigs were assembled using ABySS version 1.5.1 (BaseClear, Leiden, The Netherlands). To identify the 4HP-inducible genes, a search of the mass spectrometry-derived data against the *Arthrobacter* sp. IN13 genome was performed. The deduced amino acid sequences of the proteins encoded by the *kpi* locus were searched against database of the National Center for Biotechnology Information (NCBI) using BLAST (Basic Local Alignment Search Tool) [21]. Protein functions were assigned on the basis of a sequence similarity search against the NCBI Conserved Domain Database [22]. The phylogenetic analysis was performed with MegaX program [23].

### 2.5. Recombinant Protein Expression

*R. erythropolis* SQ1 or *E. coli* transformed with the recombinant plasmids were cultivated as described above. For protein expression and bioconversion experiments, we grew *R. erythropolis* SQ1 until the culture reached an optical density of 1.6 to 2.0 at 600 nm (OD_600_). *E. coli* BL21(DE3) was cultured aerobically at 37 °C, when an OD_600_ of 1.2 was reached, then 0.5 mM isopropyl-β-D-thiogalactopyranoside (IPTG) was added to induce the synthesis of proteins, and the culture was incubated at 20 °C for 20 h.

### 2.6. Bioconversion Reactions

***In vivo:*** For the whole-cell-based bioconversions, we grew *R. erythropolis* SQ1 cells for protein expression, which were then collected by centrifugation, washed twice with 10 mM potassium phosphate buffer (pH 7.2), and resuspended in 10 mM potassium phosphate (pH 7.2) to achieve fourfold higher cell density. Bioconversion reactions were carried out at 30 °C with shaking at 180 rpm, while monitoring the progress of conversion by the changes in the UV-VIS absorption spectrum in the 200–350 nm range. The end reaction products were analyzed by HPLC-MS.

***In vitro:*** Recombinant *E. coli* cells were grown for protein expression, harvested by centrifugation (20 min, 3220× *g*, 4 °C), then suspended in 50 mM potassium phosphate buffer (pH 7.2) and disrupted by sonication (5 min at 22 kHz, in ice-water bath, 40% of amplitude). Cell debris was removed by centrifugation at 10,000× *g* for 10 min at 4 °C. This cell-free extract was used for bioconversion reactions. The reactions were carried out at 30 °C in 96-well plates while monitoring the progress of conversion by the changes in the UV-VIS absorption spectrum in the 200–350 nm range with PowerWave XS plate reader (BioTek Instruments, Inc., Winooski, VT, USA). The end products of reactions were analyzed by HPLC-MS. The derivatization with semicarbazide was performed by adding 20-fold excess of semicarbazide to the reaction products, and the mixture was incubated for 15 min at 55 °C.

### 2.7. Bioproduction and Isolation of 3,4-Dihydroxypyridine

The bioconversion reaction was carried out in a total volume of 250 mL at 30 °C with shaking at 180 rpm. 4HP and glucose were added to the reaction mixture in portions of 10 mg and 50 mg, respectively, while monitoring the progress of conversion by the changes in the UV absorption spectrum in the 200–350 nm range. The reaction was performed for 2 days; the total amount of 4HP added was 50 mg. Bacteria were removed from the bioconversion reaction mixtures by centrifugation at 4000× *g* for 40 min, and the supernatants were evaporated under reduced pressure. The product was extracted with ethanol. For the purification of the formed 34DHP, we dissolved it in water, and then carried out reverse-phase chromatography on Grace flash cartridges C-18 (Columbia, Grace, Columbia, MD, USA) in 10% methanol. The isolated 34DHP was analyzed by HPLC-MS and NMR.

### 2.8. HPLC-MS Analysis

The samples were mixed with an equal volume of acetonitrile, vortexed for 1 min, clarified by centrifugation at 10,000× *g* for 10 min, and subjected to HPLC-MS analysis as described in [24] with the following modifications. The chromatographic separation was conducted using a C18 column, 4 × 150 mm (YMC, Kyoto, Japan) at 40 °C and a mobile phase that consisted of water with 0.1% formic acid or with 10 mM NH_4_Cl (solvent A) and acetonitrile (solvent B) delivered in gradient elution mode at a flow rate of 0.5 mL min^−1^. The elution program was used as follows: isocratic 5% B for 1 min, from 5 to 95% B over 5 min, isocratic 95% B for 2 min, from 95 to 5% B over 1 min, isocratic 5% B for 4 min.

### 2.9. Chemical Synthesis of Pyridine-3,4-Diol Hydrochloride

To 3-hydroxy-4H-pyran-4-one (1.12 g, 10 mmol) dissolved in methanol (20 mL), we added sodium hydroxide (0.44 g, 11 mmol) in water (2 mL). After the addition of benzyl bromide (1.88 g, 11 mmol), we refluxed the mixture for 8 h and allowed it to cool to room temperature. A 25% ammonia solution (40 mL) was added to the cooled solution and the resulting mixture was heated at 70 °C for 4 h. Most of the ammonia and methanol was removed in a vacuum, and the residue was filtered off, washed with water and acetone, and air-dried. The product was used as such in the following step. The foregoing crude 3-(benzyloxy)pyridin-4(1*H*)-one was refluxed with 20% HCl (40 mL) for 6 h. The solution was treated with charcoal, filtered, and excess of acid was removed in vacuum. The residue was recrystallized from propan-2-ol containing a drop of concentrated HCl to give white crystals (0.79 g, 53.5%).

^1^H NMR and ^13^C NMR spectra were recorded in DMSO-d_6_ on an Avance III 400 NMR spectrometer at 400 MHz for ^1^H and 100 MHz for ^13^C; chemical shifts are reported in parts per million relative to solvent resonance signal as an internal standard (^1^H NMR: δ (DMSO-d_6_) = 2.50 ppm; ^13^C NMR: δ (DMSO-d_6_) = 39.52 ppm).

Pyridine-3,4-diol hydrochloride. ^1^H NMR (400 MHz, DMSO-d_6_) δ 14.17 (bs, 1H), 11.31 (bs, 1H), 8.21 (d, J = 1.2 Hz, 1H), 8.16 (dd, J = 6.4, 1.2 Hz, 1H), 7.34 (d, J = 6.4 Hz, 1H). ^13^C NMR (100 MHz, DMSO-d_6_) δ 161.1, 145.1, 134.8, 127.0, 112.6 (see Figure S10).

3,4-Dihydroxypyridine. ^1^H NMR (400 MHz, DMSO-d_6_) δ 7.59 (dd, J = 6.7, 1.0 Hz, 1H), 7.51 (d, J = 1.0 Hz, 1H), 6.37 (d, J = 6.7 Hz, 1H) (see Figure S11).

### 2.10. Nucleotide Sequence Accession Number

The *Arthrobacter* sp. IN13 genome contig sequence with the 4HP degradation locus (*kpi*) was deposited in GenBank under accession no. MT469879. The *Arthrobacter* sp. IN13 16S ribosomal RNA gene sequence was deposited in GenBank under accession no. AM236151.

## 3. Results and Discussion

### 3.1. Catabolism of 4HP in Arthrobacter sp. IN13 Is Substrate-Inducible

**Figure 1:**
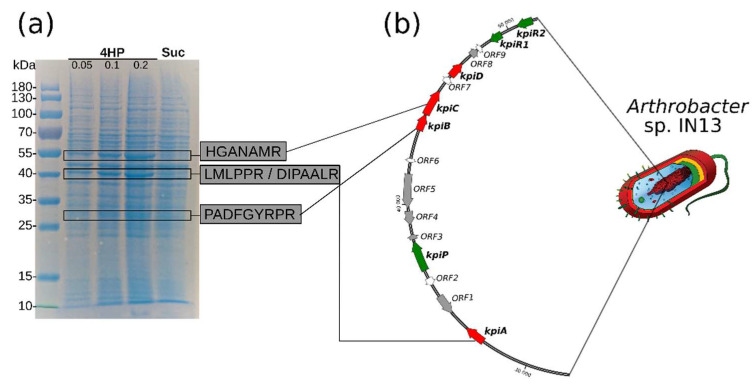
(**a**) Identification of 4-hydroxypyridine (4HP)-inducible genes in *Arthrobacter* sp. IN13. Bacteria were cultivated in minimal medium supplemented with 0.1% succinate (lane Suc) or 0.05–0.2% 4HP (lanes 4HP) as a single source of carbon. The positions of molecular mass markers are shown on the left of the gel (in kilodaltons). The peptide sequences identified by MS/MS proteomics are indicated in grey rectangles. (**b**) The organization of *Arthrobacter* sp. IN13 *kpi* gene locus and its flanking regions. Red arrows indicate genes that are known to be involved in the degradation of 4HP; green arrows indicate transport system and regulatory genes; grey arrows indicate transposases and pseudogenes; white arrows indicate hypothetical genes.

Previously we reported that *Arthrobacter* sp. IN13 is capable of using 4HP and 2HP as sources of carbon and energy [18]. To determine whether the catabolism of 4HP is inducible in this strain, we cultivated the cells of *Arthrobacter* sp. IN13 in the presence of 4HP or succinate, and the biomass was collected. The SDS-PAGE analysis of cell-free extracts of resulting biomasses showed at least three 4HP-inducible proteins (approximately 52, 42, and 30 kDa) (Figure 1), which were absent in the succinate-grown cells. In addition, bioconversion experiments using whole cells revealed that the metabolic pathway of 4HP was induced by 4HP, but not succinate (data not shown). It was concluded that catabolism of 4HP is an inducible process, and it is most likely that the three identified proteins participate in the biodegradation of 4HP; thus, they were selected for further analysis.

### 3.2. Kpi Gene Cluster Is Involved in the Degradation of 4HP

To identify genes encoding the catabolic enzymes, we extracted three 4HP-inducible proteins from SDS-PAGE and analyzed them using MS/MS sequencing (Figure 1a). All identified peptide sequences were searched against the partially sequenced genome of *Arthrobacter* sp. IN13. A contig containing all three 4HP-inducible genes was identified (MT469879). The gene cluster consisting of 16 open reading frames (ORF) was named the *kpi* locus (Figure 1b). The calculated molecular masses of *kpiC*-, *kpiA*-, and *kpiB*-encoded proteins (52.1, 41.4, 30.2 kDa, respectively) corresponded to molecular masses of proteins detected in the SDS-PAGE (52, 42, and 30 kDa, respectively) (Table 1). A comparative sequence analysis revealed that proteins encoded by *ORF1*, -*4*, and -*5* were highly similar to transposases and integrases (Table 1). Moreover, two pseudogenes (*ORF3* and *ORF8*) with incomplete gene sequences were detected in this cluster, according to their closest homologs. These data may suggest that the *kpi* locus evolved via a horizontal gene transfer, and is still in an early reorganization.

In the *kpi* cluster, both *kpiR1* and *kpiR2* genes encode hypothetical regulatory proteins and could be responsible for the expression of 4HP-inducible proteins. The amino acid sequence of KpiP shares similarities with bacterial cytosine/purine, uracil, thiamine, and allantoin permease protein family. The homologs of this protein participate in the transport of *N*-heterocyclic compounds [25,26,27]. This strongly suggests that KpiP could be responsible for the intake of 4HP. The function of four smallest genes in the *kpi* cluster (*ORF2*, *ORF6*, *ORF7*, and *ORF9*) could not be proposed by bioinformatical analysis, because no conserved domains were detected. Therefore, further investigation is required to elucidate the precise function of these proteins.

### 3.3. KpiA Encodes a 4HP-3-Monooxygenase

The majority of identified degradation pathways of hydroxypyridines begin with a (di)hydroxylation step, which is catalyzed by various mono- or dioxygenases [25,26,27]. Among genes from the *kpi* cluster, the *kpiA*-encoded protein showed the highest similarity to the enzymes of this group (Table 1). The amino acid sequence comparison using KpiA as a search query against the Pfam database placed it in Pfam01494 (FAD-binding domain 3) family. The characterized proteins that comprise the Pfam01494 family are various monooxygenases, including 2,6-dihydroxypyridine 3-monooxygenase from *Paenarthrobacter nicotinovorans* (Q93NG3), *p*-hydroxybenzoate hydroxylase from *Pseudomonas aeruginosa* (P20586), and kynurenine 3-monooxygenase from *Pseudomonas fluorescens* (Q84HF5). The phylogenetic analysis using all bacterial proteins from the Pfam01494 family with experimentally confirmed function (Figure S1) placed KpiA protein nearest to 2-heptyl-3-hydroxy-4(1H)-quinolone synthase from *Mycobacteroides abscessus* (B1MFK1) [28] (sharing 34% identity). This enzyme catalyzes the hydroxylation of 2-heptyl-4(1*H*)-quinolone, a structural homolog of 4HP, to obtain 2-heptyl-3-hydroxy-4(1*H*)-quinolone [29].

**Figure 2:**
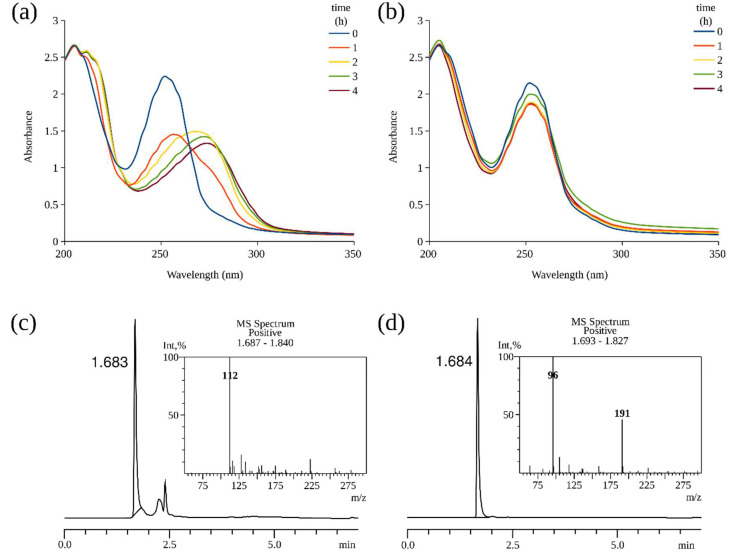
Bioconversion of 4HP in *Rhodococcus erythropolis* SQ1 transformed with recombinant pNit-*kpiA* plasmid (**a**,**c**), and wild-type *R*. *erythropolis* SQ1 (**b**,**d**). Cultures of *R*. *erythropolis* SQ1 were incubated in potassium phosphate buffer supplemented with 1 mM 4HP, and the UV-VIS absorption spectra were recorded (**a**,**b**). The lines represent the progress of the reaction (time in hours). The end reaction products (after 4 h) were analyzed by HPLC-MS (**c**,**d**).

On the basis of these data, we hypothesized that the 4HP-inducible protein KpiA is a hypothetical monooxygenase and performs an initial hydroxylation of 4HP at the third position. To confirm such activity, we tried to express the *kpiA* gene in *E. coli*. However, all our attempts to obtain a functional KpiA protein in *E. coli* cells failed due to the aggregation of the insoluble protein. To solve this problem, *Rhodococcus erythropolis* SQ1 cells, which are unable to metabolize 4HP, were tested as an alternative host for expression of the recombinant protein. Thus, the *kpiA* gene was cloned to a broad host range vector (pNitQC1) to obtain pNit-*kpiA* construct. After transformation with pNit-*kpiA* plasmid, *R*. *erythropolis* SQ1 cells acquired the ability to convert 4HP into a new product according to the UV-VIS analysis (Figure 2). In contrast, the non-transformed cells did not change 4HP even after a prolonged incubation. As seen in Figure 2, *R*. *erythropolis* SQ1 cells harboring pNit-*kpiA* plasmid converted 4HP to a metabolite with a UV-VIS absorbance peak at 276 nm. The bathochromic shift of 24 nm suggested that hydroxylation of the pyridine ring took place. The HPLC-MS analysis of the reaction mixture showed one main product. The protonated molecular ion with *m/z* = 112 corresponded to an increase in the mass of the substrate by 16 Da, implying the incorporation of one oxygen atom (Figures S2 and S3). The product gave a distinct purple color when mixed with FeCl_3_, which is characteristic of 3,4-dihydroxypyridine (34DHP) [13]. To further identify the product of KpiA monooxygenase, we used *R*. *erythropolis* SQ1 cells carrying pNit-*kpiA* plasmid to convert a larger amount of 4HP, as described in the Materials and Methods section. After the purification of the compound, we performed HPLC-MS and ^1^H NMR analysis. The product of hydroxylation of 4HP by KpiA was identified as 34DHP according to the NMR spectra (^1^H NMR (DMSO-d_6_, ppm): δ = 7.50 (dd, J = 6.8, 1.5 Hz); δ 7.39 (s); δ 6.18 (d, J = 6.8 Hz)). In addition, the 34DHP was synthesized chemically, and its characteristics were identical to the product of the KpiA-catalyzed reaction. In summary, these results were in line with the previous studies on *Agrobacterium* sp. cells, in which the degradation of 4HP also started with a hydroxylation reaction at the third position of the pyridine ring [13].

### 3.4. KpiC Is an Extradiol Dioxygenase

**Figure 3:**
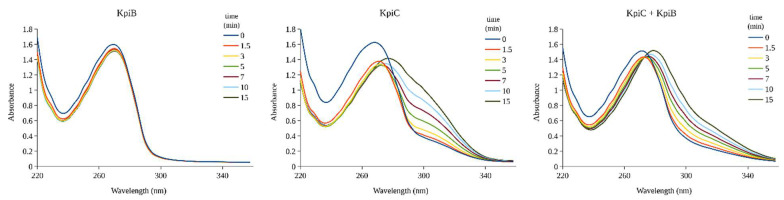
Bioconversion of 34DHP with the cell-free extracts of *Escherichia*. *coli* BL21 (DE3) expressing recombinant proteins (KpiB, KpiC, or both). The cell-free extracts were incubated in potassium phosphate buffer supplemented with 0.5 mM 34DHP, and the UV-VIS absorption spectra were recorded. The lines represent the progress of the reaction (time in minutes).

The enzymatic activity of a 34DHP dioxygenase was previously determined in the 4HP-degrading bacteria *Agrobacterium* sp. NCIB 10413 [14] and anticipated in mimosine-metabolizing bacteria *Rhizobium* sp. TAL1145 [16]. On the basis of sequence analysis, we annotated protein PydA from *Rhizobium* sp. TAL1145 as a 34DHP dioxygenase, belonging to the Pfam02900 family (the catalytic LigB subunit of aromatic ring-opening dioxygenases). However, the analysis of the *kpi* cluster did not reveal any homolog of aromatic ring-opening dioxygenases, and only a low-level identity (≈20%) between PydA and KpiC was detected. Moreover, according to the phylogenetic analysis, both KpiC and KpiB (another two 4HP-inducible proteins) belonged to different families of hydrolases, but not oxygenases. To elucidate the fate of 34DHP in *Arthrobacter* sp. IN13 during degradation of 4HP, we cloned both *kpiB* and *kpiC* genes to the expression vectors (Table S1), and the appropriate individual proteins were successfully overproduced in *E*. *coli* BL21(DE3). The cell-free extract containing the recombinant KpiB or KpiC enzyme was mixed with chemically synthesized 34DHP, and the progress of the reaction was monitored using UV-VIS spectroscopy. As a result, the KpiC protein, but not KpiB, was able to catalyze the conversion of the added substrate (Figure 3). HPLC-MS analysis of the reaction mixture revealed the presence of one product with *m/z* 143 (Figure S4). Thus, the molecular mass of the product was 32 Da higher than that of 34DHP. Furthermore, the reaction product reacted with semicarbazide (Figure S5), indicating the presence of a keto or an aldehyde group in the structure. In summary, these results suggested that KpiC catalyzed an extradiol ring-cleavage of 34DHP through the addition of two oxygen atoms, leading to the formation of a new metabolite. We were not able to purify the reaction product of the conversion of 34DHP by KpiC. A previous study postulated that the ring cleavage of 34DHP occurred between C2 and C3 by the formation of 3-(*N*-formyl)-formiminopyruvate (3NfFIP) as the product [14], although it was not isolated or detected. Our data are in line with the published data; however, an unambiguous validation of the structure of the product of KpiC-catalyzed reaction requires additional studies.

**Figure 4:**
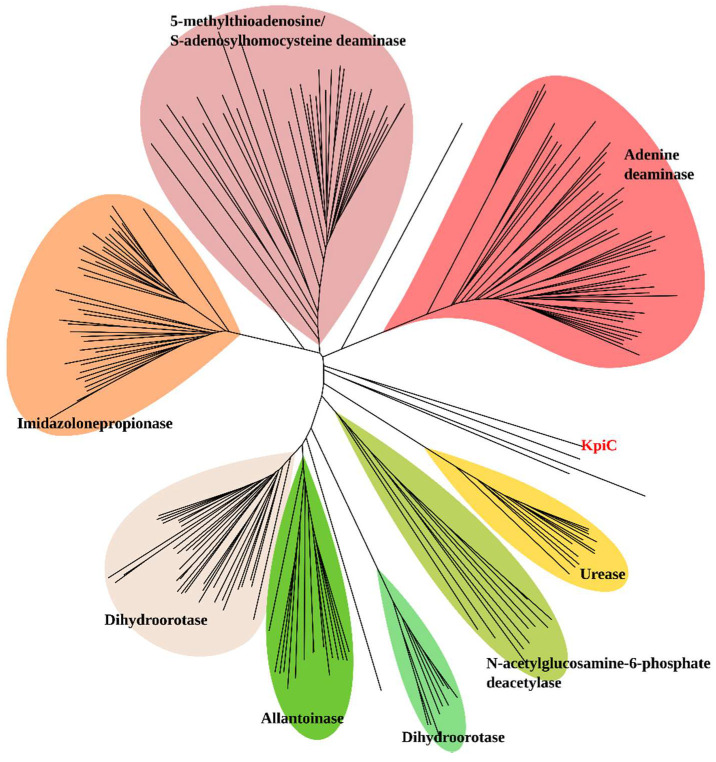
Phylogenetic analysis of KpiC in comparison with the selected enzymes from amidohydrolase family (Pfam01979). See Table S2 for the accession numbers of the enzymes used in this phylogenetic analysis.

To elucidate the phylogenetic position of KpiC in detail, we performed a BLAST search against the non-redundant protein sequences of the NCBI database. It identified KpiC as a member of the amidohydrolase-1 superfamily (Pfam01979). A phylogenetic analysis of the reviewed protein sequences from the UniProt database belonging to the Pfam01979 revealed that KpiC did not cluster with any well-characterized enzyme group, such as imidazolone propionases, 5-methyltioadenosine/S-adenosylhomocysteine deaminases, adenine deaminases, ureases (alpha subunit), *N*-acetylglucosamine-6-phosphate deacetylases, dihydroorotases, or allantoinases (Figure 4). The closest homologs of KpiC were an uncharacterized protein (P9WL22), a hypothetical urease (beta subunit) (P50045), and enamidase (Q0QLE9). At the time of the study, only the enamidase from *Eubacterium barkeri* had an experimentally confirmed function and showed 28% identity with KpiC protein. This enzyme participates in the catabolic pathway of nicotinate, and catalyzes the hydrolysis of 6-oxo-1,4,5,6-tetrahydronicotinate to ammonia and (*S*)-2-formylglutarate [30]. Other well-characterized enzymes from the Pfam01979 superfamily are metallo-dependent hydrolases. The active site of these enzymes usually contains two divalent metal ions, such as Ni^2+^, Fe^2+^, Zn^2+^, Co^2+^, or Mn^2+^. However, several enzymes, e.g., Zn^2+^-dependent *N*-acetylglucosamine-6-phosphate deacetylase from *E*. *coli* (P0AF18) [31] and dihydroorotase from *Aquifex aeolicus* (O66990) [32], as well as Fe^2+^-dependent atrazine chlorohydrolase from *Pseudomonas* sp. ADP (P72156) [33], are active with just one metal ion per subunit. Remarkably, the extradiol 2,5-dihydroxypyridine 5,6-dioxygenases NicX and HpdF, which participate in the catabolic conversion of nicotinic acid and 2-hydroxypyridine, respectively, do not show any significant similarities to the known dioxygenases, but are rather homologous to metallo-dependent aminopeptidases [27,34]. The 3D model of NicX proposes that one of the metal-binding sites is lost, and the experiments confirm that a single Fe^2+^ ion per protein subunit is sufficient for the O_2_-dependent ring opening of 25DHP [34]. In conclusion, the accumulated data strongly suggest that KpiC is a novel dioxygenase that catalyzes the ring opening of 34DHP.

### 3.5. KpiB Hydrolyzes 3-(N-formyl)-Formiminopyruvate to 3-Formylpyruvate

**Figure 5:**
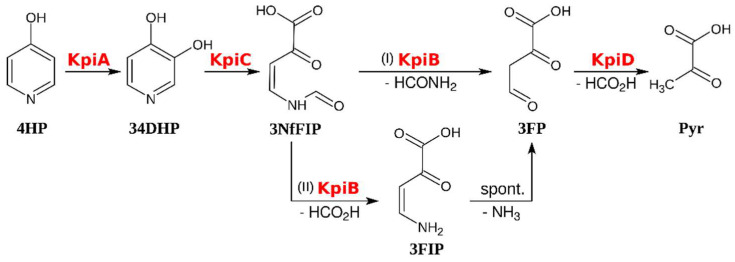
Proposed catabolic pathway of 4HP in *Arthrobacter* sp. IN13. 4-Hydroxypyridine (4HP), 3,4-dihydroxypyridine (34DHP), 3-(*N*-formyl)-formiminopyruvate (3NfIP), 3-formiminopyruvate (3FIP), 3-formylpyruvate (3FP), pyruvate (Pyr).

The third 4HP-inducible protein detected in *Arthrobacter* sp. IN13 cells was the product of the *kpiB* gene. To check whether KpiB participates in the metabolism of 4HP, we overexpressed the *kpiB* gene from plasmid pET-*kpiB*. The recombinant KpiB protein migrated in SDS-PAGE as an intense 30 kDa band that agreed with a molecular weight predicted for the product of the *kpiB* gene (29.5 kDa). As we were unable to purify the reaction product of the KpiC-catalyzed reaction, we used both proteins (KpiC and KpiB) in a one-pot reaction. The UV-VIS analysis (Figure 3) showed that the cell-free extract containing both KpiC and KpiB proteins converted 34DHP to a novel product distinct from one produced when the cell-free extract harbored the enzyme KpiC only. In addition, the HPLC-MS analysis of the reaction mixture revealed that both 34DHP and the product of the KpiC-catalyzed reaction were fully consumed (Figure S6), but the MS-based identification of the newly formed compound was inconclusive. However, after derivatization with semicarbazide, we were able to determine that the molecular mass of this metabolite was 116 Da (Figure S7). Remarkably, the end product of two reactions was coupled with two molecules of semicarbazide after derivatization. These results suggested that the formula of this new compound most likely corresponded to 3-formylpyruvate (3FP), an intermediate proposed in the degradation of 4HP by *Agrobacterium* sp. 35S [14] and in the metabolism of mimosine by *Rhizobium* sp. strain TAL1145 as well [16]. There are two possible ways how the hydrolysis of 3NfFIP to 3FP can occur. It might be completed in one step directly releasing formamide or in two consecutive reactions through intermediate 3-formiminopyruvate (3FIP) (Figure 5).

The amino acid sequence of KpiB showed similarity to members of the α/β hydrolase fold superfamily (Figure S8). This superfamily contains enzyme activities of apparently diverse functions, e.g., acetylcholinesterase, dienelactone hydrolase, lipase, thioesterase, serine carboxypeptidase, proline iminopeptidase, proline oligopeptidase, haloalkane dehalogenase, haloperoxidase, epoxide hydrolase, hydroxynitrile lyase, and others [35]. All members of the α/β hydrolase fold superfamily depend on a nucleophile-His-Acid catalytic triad to efficiently operate on various substrates. The *N*-formylmaleamate deformylase (nicD) from *Pseudomonas putida* KT2440 involved in nicotinic acid degradation also belongs to α/β hydrolase fold superfamily [34], although it has low identity with KpiB protein (21%). The deformylation of 3-(*N*-formyl)-formiminopyruvate was proposed in the degradation of 4HP by *Agrobacterium* 35S [14] and in the metabolism of mimosine by *Rhizobium* sp. strain TAL1145 as well [16]. Since enamines are unstable in water and spontaneously transform to ketones or aldehydes, special efforts should be made to confirm a way by which the hydrolysis of 3-formiminopyruvate to 3-formylpyruvate proceeds in the presence of KpiB. This requires a separate investigation.

It has been proposed that the metabolism of 4HP in *Agrobacterium* sp. 35S ends with the hydrolysis of 3-formylpyruvate to formate and pyruvate [14]. This step in *Arthrobacter* sp. IN13 is probably performed by the KpiD protein. According to the BLAST homology search and phylogenetic analysis, KpiD belongs to the fumarylacetoacetate hydrolase family (Pfam01557) (Figure S9). Members of this family often participate in the final steps of the metabolism of aromatic compounds, e.g., 2-keto-4-pentenoate hydratase (MhpD) is involved in the phenylpropionic acid pathway of *E. coli* [36], 5-oxo-pent-3-ene-1,2,5-tricarboxylic acid decarboxylase/isomerase (HpcE) participates in the 4-hydroxyphenylacetic acid degradation pathway of *E. coli* [37], and fumarylacetoacetase catalyzes the hydrolytic cleavage of a C-C bond in fumarylacetoacetate to yield fumarate and acetoacetate as the final step in phenylalanine and tyrosine degradation in humans [38].

All our attempts to obtain a functional recombinant KpiD protein in *E*. *coli* cells failed due to the formation of insoluble protein aggregates. Moreover, the expression of KpiD in *R*. *erythropolis* SQ1 was not detected at all. Hence, the experimental confirmation of the function of the KpiD protein should await new and improved expression hosts.

## 4. Conclusions

The *kpi* gene locus containing genes that are responsible for the catabolism of 4HP in *Arthrobacter* sp. IN13 was identified and characterized. The first reaction of 4HP degradation is catalyzed by a regiospecific flavin-dependent monooxygenase KpiA, and the product of this reaction is 34DHP. During the second step of the enzymatic degradation of 4HP, a fission of the pyridine ring is performed by a novel extradiol dioxygenase KpiC, which belongs to the amidohydrolase family. It can be speculated that the active site residues of the enzyme are probably rearranged to be able to catalyze an oxygenolytic ring fission reaction. The 3-(*N*-formyl)-formiminopyruvate formed is further converted to 3-formylpyruvate by KpiB hydrolase. It is more likely that KpiB is a deformylase, and 3-formiminopyruvate spontaneously hydrolyzes to 3-formylpyruvate. We propose that the last step in the metabolism of 4HP is executed by KpiD, which is homologous to fumarylacetoacetate hydrolases. In summary, our results render new fundamental knowledge about the degradation pathways of *N*-heterocyclic aromatic compounds and enrich the pool of enzymes that may be used in biocatalysis. Further studies are necessary for the detailed characterization of newly identified enzymes and their practical applications.

## Figures and Tables

**Table 1:** Functional Annotations of Hypothetical Kpi Proteins.

Protein	Size (Amino Acids)	Putative Function	Superfamily (Specific Hit/Conserved Domain)	Accession No.
**KpiA**	374	Monooxygenase	FAD-binding domain_3	pfam01494
**ORF1**	363	Transposase	rve superfamily	cl21549
**ORF2**	135	Hypothetical	CBS domain-containing protein	cd02205
**KpiP**	533	Permease	SLC5-6-like-sbd superfamily	cd11484
**ORF3**	84	Pseudogene	Incomplete; transposase and inactivated derivatives	
**ORF4**	273	Transposase	DNA replication protein DnaC	COG1484
**ORF5**	590	Transposase	IS21 family transposase	COG4584
**ORF6**	66	Hypothetical	No putative conserved domains have been detected	
**KpiB**	295	Hydrolase	Alpha/beta hydrolase_1 family	pfam00561
**KpiC**	475	Dioxygenase	Amidohydrolase_1 family	pfam01979
**ORF7**	108	Hypothetical	No putative conserved domains have been detected	
**KpiD**	288	FAA hydrolase	Fumarylacetoacetate hydrolase family	pfam01557
**ORF8**	135	Pseudogene	Incomplete; missing N-terminus; MopB_CT superfamily	cl09929
**ORF9**	74	Hypothetical	No putative conserved domains have been detected	
**KpiR1**	224	Regulatory protein	DNA-binding transcriptional regulator, FadR family	COG2186
**KpiR2**	294	Regulatory protein	AraC-type DNA-binding domain	COG2207

## Supplementary Materials

The following are available online at https://www.mdpi.com/2076-2607/8/6/888/s1, Table S1: Bacterial strains, plasmids, and primers used in this study. Figure S1: The phylogenetic analysis for KpiA protein. Figure S2: HPLC-MS analysis of the bioconversion reaction mixture of 4HP with *R. erythropolis* SQ1 whole-cells. Figure S3: HPLC-MS analysis of the bioconversion reaction mixture of 4HP with *R. erythropolis* SQ1 transformed with pNit-*kpiA* plasmid. Figure S4: HPLC-MS analysis of the reaction mixture of 34DHP with cell-free extract of *E. coli* BL21(DE3) harboring pET-*kpiC* plasmid. Figure S5: HPLC-MS analysis of the reaction mixture of 34DHP with cell-free extracts of *E. coli* BL21(DE3) harboring pET-*kpiC* plasmid and derivatized with semicarbazide. Table S2: The selected enzymes from amidohydrolase family (Pfam01979) for phylogenetic analysis of KpiC. Figure S6: HPLC-MS analysis of the reaction mixture of 34DHP with cell-free extract of *E. coli* BL21(DE3) harboring pET-*kpiC* and pET-*kpiB* plasmids. Figure S7: HPLC-MS analysis of the reaction mixture of 34DHP with cell-free extract of *E. coli* BL21(DE3) harboring pET-*kpiC* and pET-*kpiB* plasmids and derivatized with semicarbazide. Figure S8: The phylogenetic analysis for KpiB protein. Figure S9: The phylogenetic analysis for KpiD protein. Figure S10: NMR spectra of chemically synthesized pyridine-3,4-diol hydrochloride. Figure S11: NMR spectra of chemically synthesized 3,4-dihydroxypyridine.
