# Supplementary material for: Biochemical and Genetic Analysis of 4-Hydroxypyridine Catabolism in Arthrobacter sp. Strain IN13
Source: Microorganisms. 2020 Jun 12;8(6):888. doi: 10.3390/microorganisms8060888 (PMC7356986; doi:10.3390/microorganisms8060888)
Supplement: Supplementary file 1 [file microorganisms-08-00888-s001.pdf]

Supplementary Material for

Biochemical and genetic analysis of 4-hydroxypyridine catabolism in *Arthrobacter* sp. strain IN13

Justas Vaitekūnas [1*], Renata Gasparavičiūtė [1], Jonita Stankevičiūtė [1], Gintaras Urbelis [2], and Rolandas Meškys [1]

[1]Department of Molecular Microbiology and Biotechnology, Institute of Biochemistry, Life Sciences Center, Vilnius University, Saulėtekio al. 7, Vilnius, LT-10257, Lithuania.
[2]Department of Organic Chemistry, Center for Physical Sciences and Technology, Akademijos 7, LT-08412 Vilnius, Lithuania.

*Correspondence: justas.vaitekunas@bchi.vu.lt

Table S1. Bacterial strains, plasmids, and primers used in this study

Strain, plasmid, or primer	Genotype or relevant characteristics, relevant properties and cloning strategies, or sequence (5'–3')	Reference or source
**Strains**		
Arthrobacter sp. IN13	4HP-degrading bacterium	[2]
R. erytropolis SQ1	Mutant of *Rhodococcus erythropolis* strain ATCC 4277-1 with increased transformability	[3]
E. coli DH5α	Host for cloning and plasmid isolation	Novagen, Germany
E. coli BL21(DE3)	Host for protein expression and bioconversion	Novagen, Germany
**Plasmids**		
pTZ57R/T	Ap^r *ori* ColE1 *lacZα*, high copy-no. cloning vector	Thermo Fisher Scientific, Lithuania
pET-21b	pBR322-derived ColE1, T7 *lac* promoter, Ap^r	Novagen, Germany
pET-28b	pBR322-derived ColE1, T7 *lac* promoter, Km^r	Novagen, Germany
pNitQC1	*E. coli-Rhodococcus* shuttle vector for constitutive expression, Chl^r *repAB*	[1]
pNit-*kpiA*	The kpiA gene was amplified by PCR using genomic DNA from *Arthrobacter* sp. IN13 and primers kpiAF and kpiAR, digested with NdeI and XhoI, and cloned into the corresponding sites of the pNitQC1 vector	This study
pET-*kpiB*	The kpiB gene was amplified by PCR using genomic DNA from *Arthrobacter* sp. IN13 and primers kpiBF and kpiBR, digested with NdeI and XhoI, and cloned into the corresponding sites of the pET-21b vector	This study
pET-*kpiC*	The kpiC gene was amplified by PCR using genomic DNA from *Arthrobacter* sp. IN13 and primers kpiCF and kpiCR, digested with NdeI and HindIII, and cloned into the corresponding sites of the pET-28b vector	This study
**Primers**		
kpiAF	AGATATA<u>CATATG</u>AGAACTGCTGAAATTG	This study
kpiAR	GTGGTG<u>CTCGAG</u>CTAGGCCCCGGCTG	This study
kpiBF	AGGAGATATA<u>CATATG</u>ACGGGAGCCTA	This study
kpiBR	GTGGTG<u>CTCGAG</u>TCAATTGCCTCCTATGG	This study
kpiCF	GAAGGAGATATA<u>CATATG</u>CCGAAGTATTTG	This study
kpiCR	GAGAGATCT<u>AAGCTT</u>CATTCTCCCACTGG	This study

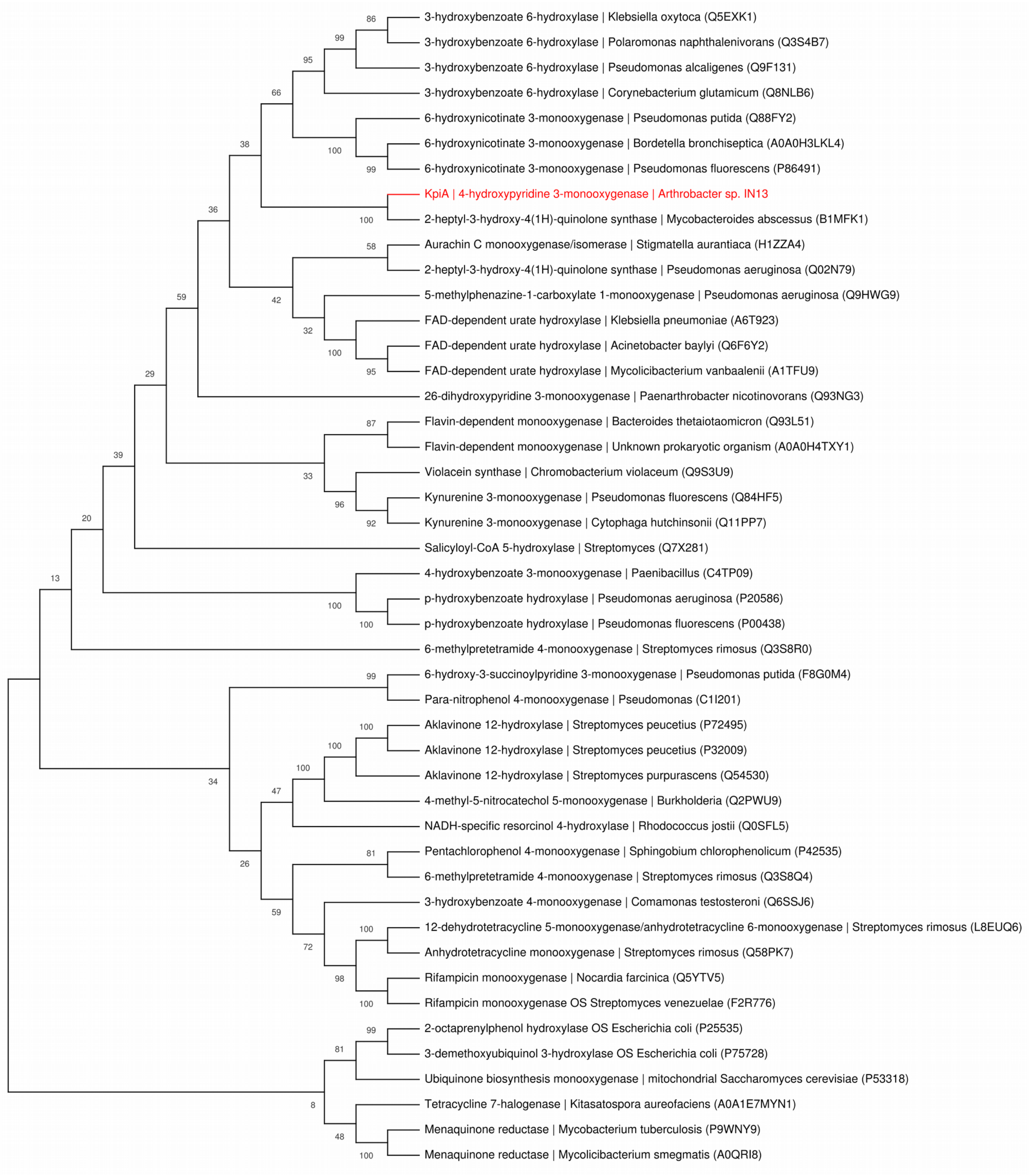

Figure S1. The phylogenetic analysis for KpiA in comparison with selected enzymes from FAD binding domain_3 family (pfam01494). Proteins were selected from UniProtKB database using determinants: pf01494 and bacteria and annotation:(type: "catalytic activity" evidence:"Inferred from experiment") and reviewed:yes. The evolutionary history was inferred by using the Maximum Likelihood method and JTT matrix-based model [4]. The bootstrap consensus tree inferred from 300 replicates [5] is taken to represent the evolutionary history of the taxa analyzed [5]. Branches corresponding to partitions reproduced in less than 50% bootstrap replicates are collapsed. The percentage of replicate trees in which the associated taxa clustered together in the bootstrap test (300 replicates) are shown next to the branches [5]. Initial tree(s) for the heuristic search was obtained automatically by applying Neighbor-Join and BioNJ algorithms to a matrix of pairwise distances estimated using the JTT model, and then selecting the topology with superior log likelihood value. This analysis involved 46 amino acid sequences. There were a total of 808 positions in the final dataset. Evolutionary analyses were conducted in MEGA X [6].

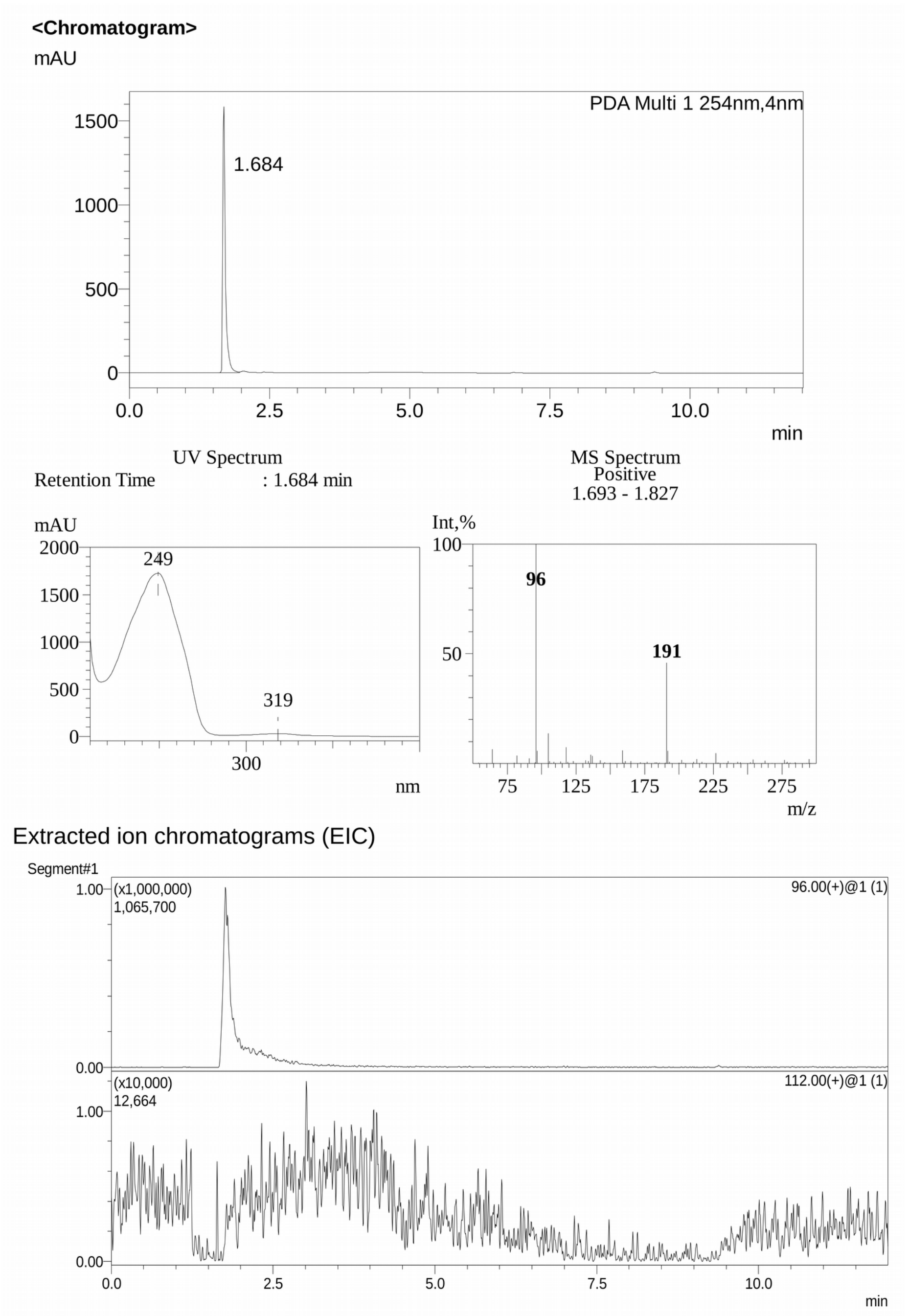

Figure S2. HPLC-MS analysis of the bioconversion of 4HP (1 mM) performed with whole-cells of *R. erythropolis* SQ1 (wild-type). The bioconversion was carried out at 30 °C for 4 hours. EIC of m/z 96 $[M+H]^+$ corresponds to 4HP and EIC of m/z 112 $[M+H]^+$ corresponds to 34DHP.

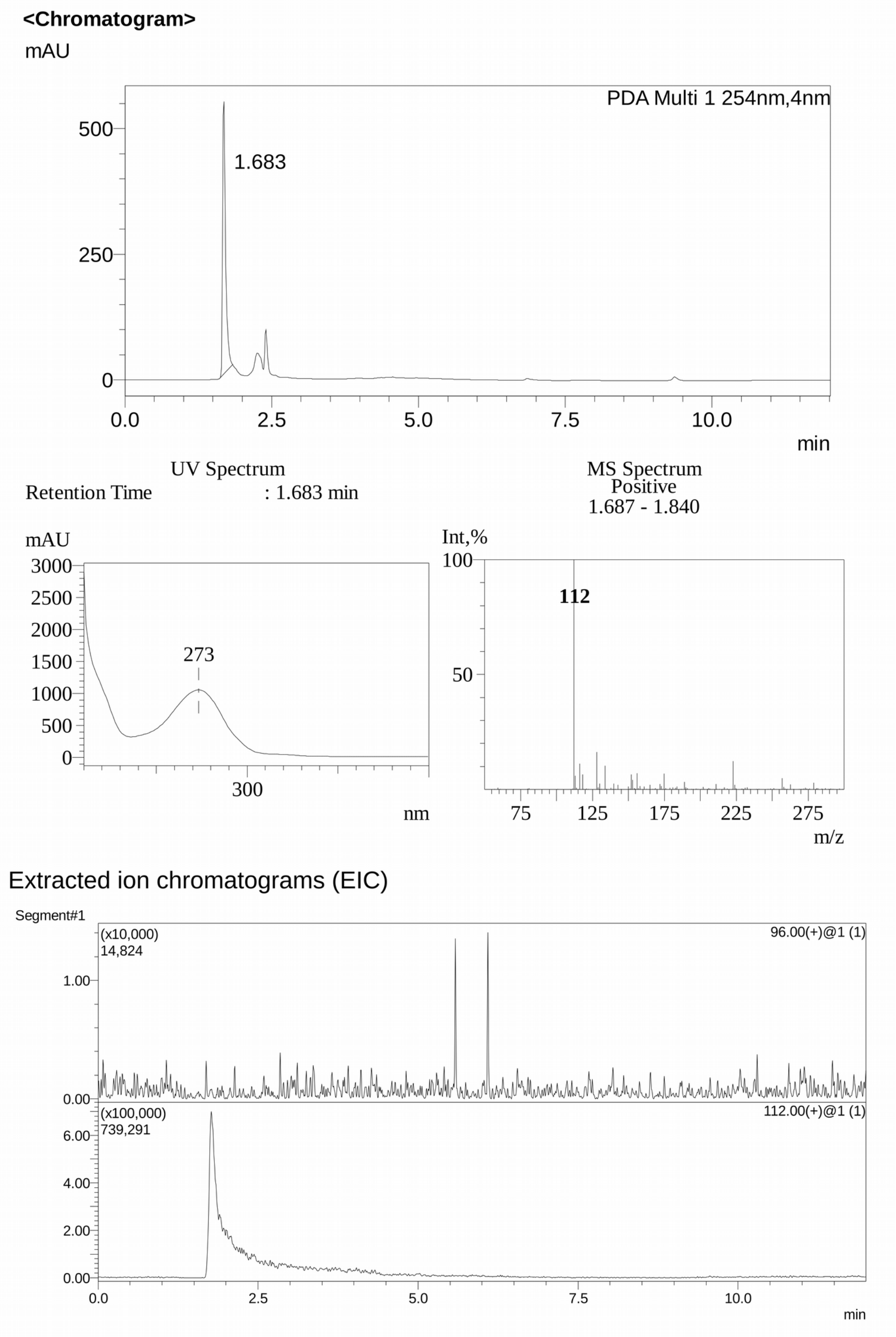

Figure S3. HPLC-MS analysis of the bioconversion of 4HP (1 mM) performed with whole-cells of *R. erythropolis* SQ1 harboring pNit-*kpiA* plasmid. The bioconversion was carried out at 30 °C for 4 hours. EIC of m/z 96 $[M+H]^+$ corresponds to 4HP and EIC of m/z 112 $[M+H]^+$ corresponds to 34DHP.

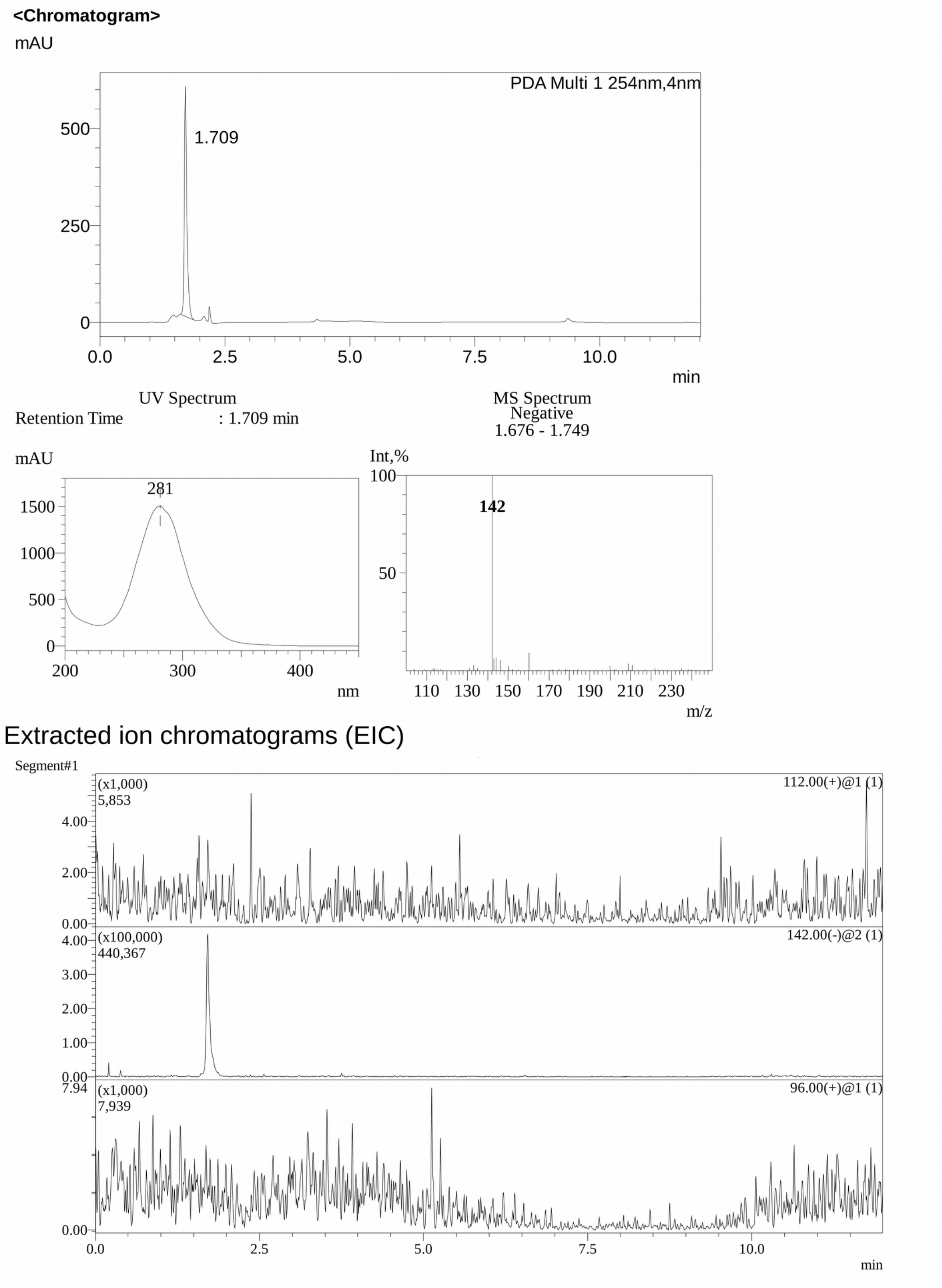

Figure S4. HPLC-MS analysis of the bioconversion of 34DHP performed with cell-free extract of *E. coli* BL21(DE3) harboring pET-*kpiC* plasmid. The bioconversion was carried out at 30 °C for 20 minutes. EIC of m/z 112 $[M+H]^+$ corresponds to 34DHP, EIC of m/z 142 $[M-H]^-$ corresponds to 3-(*N*-formyl)-formiminopyruvate, and EIC of m/z 96 $[M+H]^+$ corresponds to 4HP.

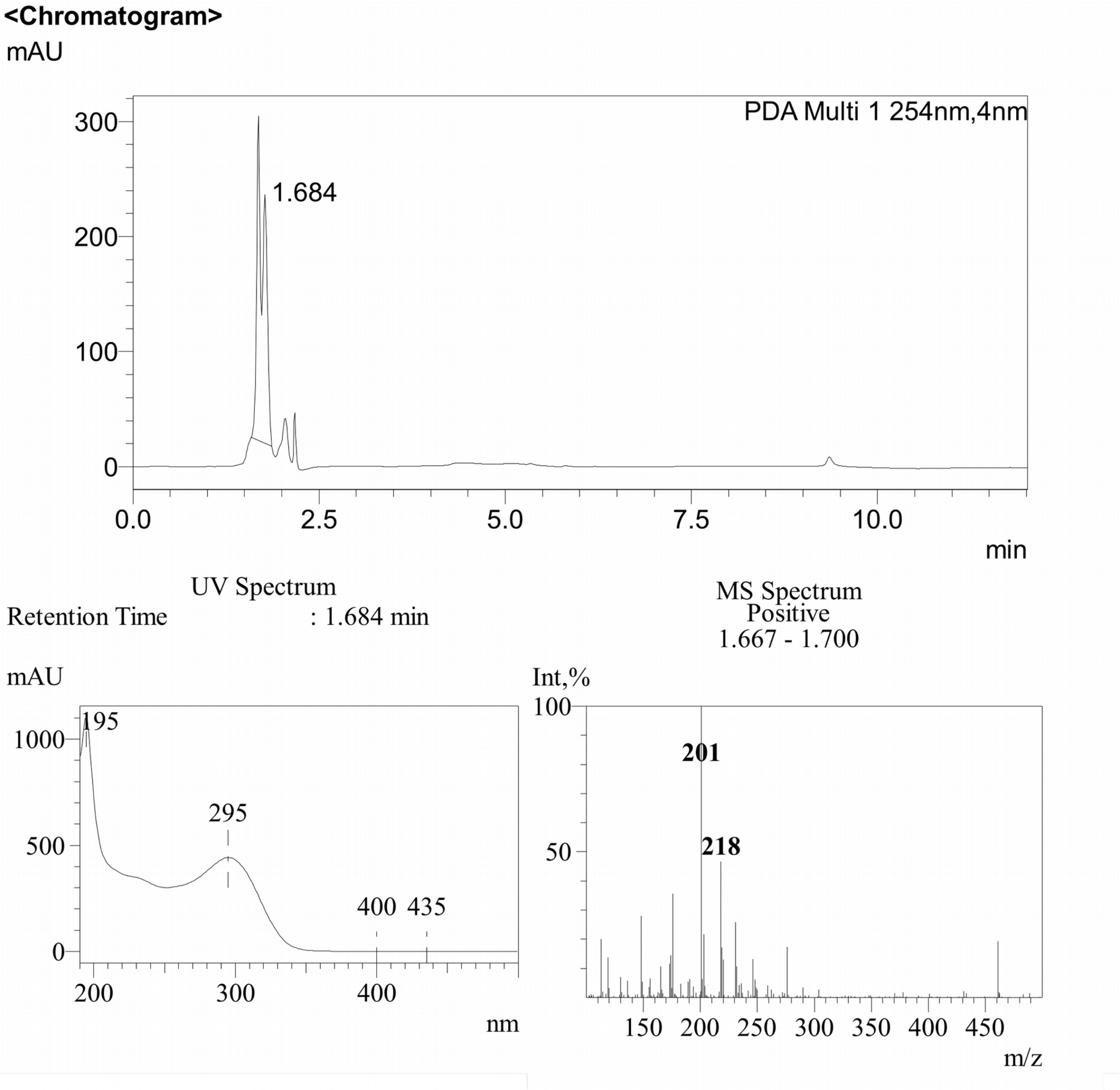

Figure S5. HPLC-MS analysis of the semicarbazide derivatized 34DHP bioconversion product. The bioconversion was carried out with cell-free extracts of *E. coli* BL21(DE3) harboring pET-*kpiC* plasmid at 30 °C for 20 minutes, and then the reaction mixture was derivatized with semicarbazide. m/z 201 $[M+H]^+$ represents 3-(*N*-formyl)-formiminopyruvate derivatized with semicarbazide, m/z 218 $[M+NH_4]^+$ is an NH_4^+ adduct of 201.

Table S2. The selected enzymes from amidohydrolase family (Pfam01979) for phylogenetic analysis of KpiC. Proteins were selected from UniProtKB database using determinants: Pfam01979 and bacteria and reviewed and clustered with identity of 0,5. The accession numbers of the selected enzymes used in this phylogenetic analysis. The evolutionary history was inferred using the Neighbor-Joining method [7]. The optimal tree from bootstrap test (1000 replicates) is shown [5]. The tree is drawn to scale, with branch lengths expressed in the same units as those of the evolutionary distances used to infer the phylogenetic tree. The evolutionary distances were computed using the Poisson correction method [8] and are expressed in the units of the number of amino acid substitutions per site. The proportion of sites where at least 1 unambiguous base is present in at least 1 sequence for each descendent clade is shown next to each internal node in the tree. This analysis involved 206 amino acid sequences. Evolutionary analyses were conducted in MEGA X [6].

A0KF84	B2VC08	O66851	P9WHL2	Q2IIB0	Q5E0C3	Q82LL4	Q8ZQR2
A0KYQ5	B3E3I1	O66990	P9WL22	Q2LTB7	Q5GSJ0	Q835Z6	Q92YE0
A0LFB5	B4UEL6	O69809	Q01V55	Q2LUH4	Q5SK67	Q837K0	Q972W0
A0LK22	B5YDN9	O86508	Q01W05	Q2LVI3	Q5WBJ6	Q84F86	Q97KN0
A0LKV6	B5YLB7	P06204	Q03HB2	Q2RJW1	Q5WCP8	Q88SR1	Q97LN7
A0LMI3	B7N966	P0AF19	Q04HC1	Q2S006	Q5WD17	Q891Y7	Q9CCR4
A0LZE0	B7NW14	P0CI72	Q08716	Q2YPD5	Q5WJ39	Q89GV2	Q9CGM7
A0QRN6	B8CX03	P17086	Q087W5	Q2YQD8	Q5ZXM0	Q89H53	Q9FCD3
A1S1N6	B8E183	P18314	Q0AFI1	Q317F9	Q65LN0	Q89QG3	Q9HU91
A3DEQ2	B8FLT9	P25995	Q0AYV2	Q38V07	Q67JH7	Q8A4B1	Q9I6Z0
A4J556	B8GA45	P39377	Q0QLE9	Q38X26	Q67NQ5	Q8CUS7	Q9KAH8
A4J872	B8HA07	P39761	Q0SLI9	Q38ZJ0	Q6ANH1	Q8EB40	Q9KC82
A4XJI3	B9MS22	P41020	Q141F5	Q39US3	Q6AS41	Q8KDK5	Q9KF46
A6GYI0	C0QJ54	P42084	Q15YC5	Q3AC01	Q6FD29	Q8PLZ7	Q9KF49
A6LG60	C5BF52	P42358	Q1AVY7	Q3AC64	Q6MJP9	Q8PUQ3	Q9KZ78
A6Q234	C5BSJ0	P42906	Q1AYH1	Q3AE81	Q6MJY0	Q8PVF4	Q9RKU5
A6T7D6	C5CFQ7	P50045	Q1AZ25	Q3AE90	Q72B14	Q8R9L4	Q9RV76
A6VXW9	C5CI63	P50047	Q1D4A5	Q3IJC9	Q72EX7	Q8R9R6	Q9RVC3
A7GA53	O25045	P57416	Q1G825	Q3J770	Q73RN8	Q8RFG1	Q9RW45
A8GAT0	O25046	P58080	Q1MR44	Q3Z8C6	Q7CS13	Q8TNH5	Q9RYP0
A9BIU9	O31196	P72156	Q1Q9E2	Q45515	Q7CUX4	Q8U8Z6	Q9RYX4
B0KCB7	O32137	P76641	Q1WTM3	Q46812	Q7CXF0	Q8VTT5	Q9RZ05
B1HR71	O32445	P77884	Q21IS0	Q51551	Q7P192	Q8XAC3	Q9X034
B2KCN5	O34450	P81006	Q24PT9	Q52725	Q7UNR2	Q8XKX4	
B2TNG1	O34909	P95442	Q24R88	Q59699	Q81BX6	Q8Y6E5	
B2V5I6	O52063	P96166	Q28VK7	Q59712	Q81F14	Q8YD09	

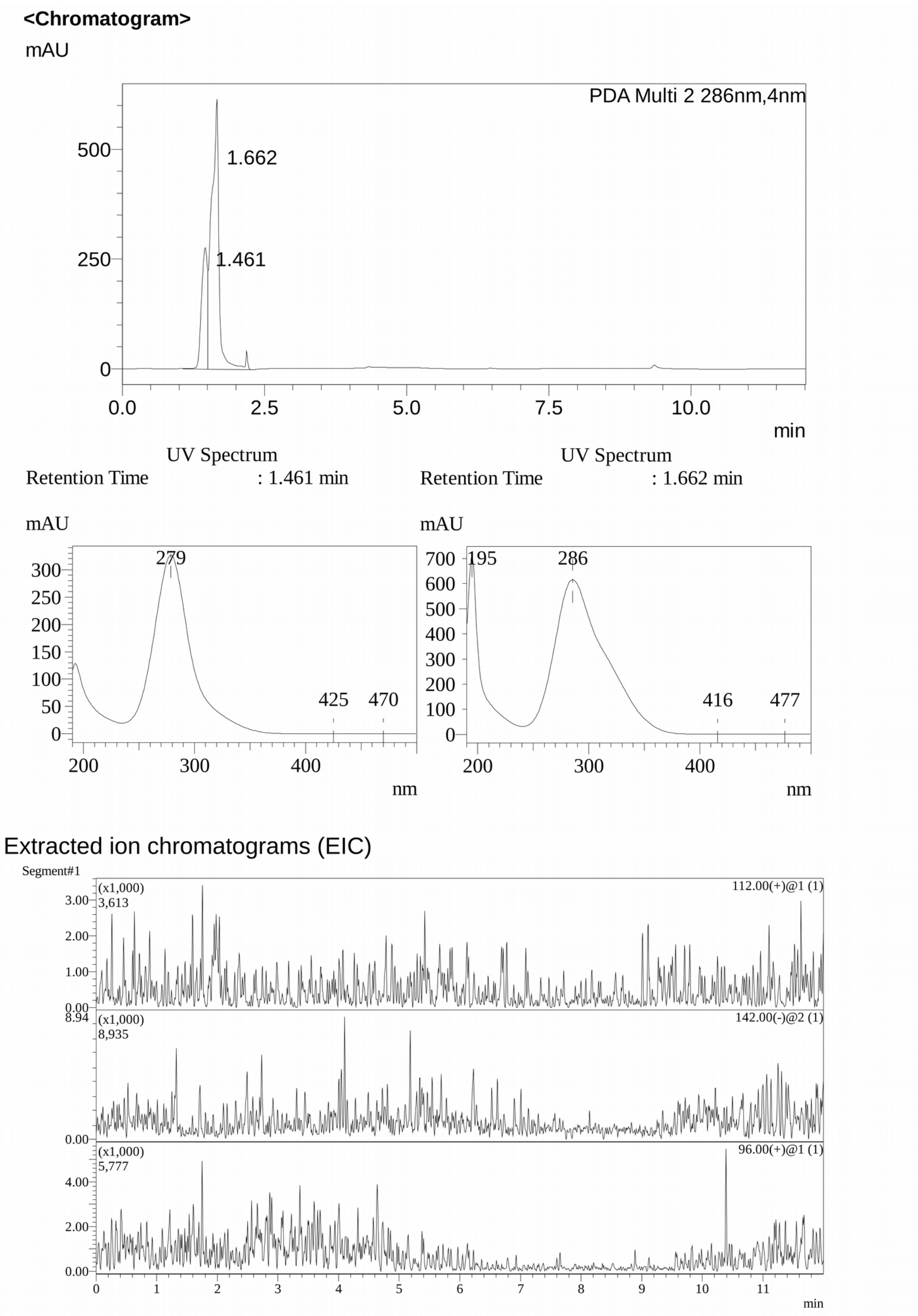

Figure S6. HPLC-MS analysis of the bioconversion of 34DHP performed with cell-free extract of *E. coli* BL21(DE3) harboring pET-*kpiC* and pET-*kpiB* plasmids. The bioconversion was carried out at 30 °C for 20 minutes. EIC of m/z 112 $[M+H]^+$ corresponds to 34DHP, EIC of m/z 142 $[M-H]^-$ corresponds to 3-(*N*-formyl)-formiminopyruvate, and EIC of m/z 96 $[M+H]^+$ corresponds to 4HP.

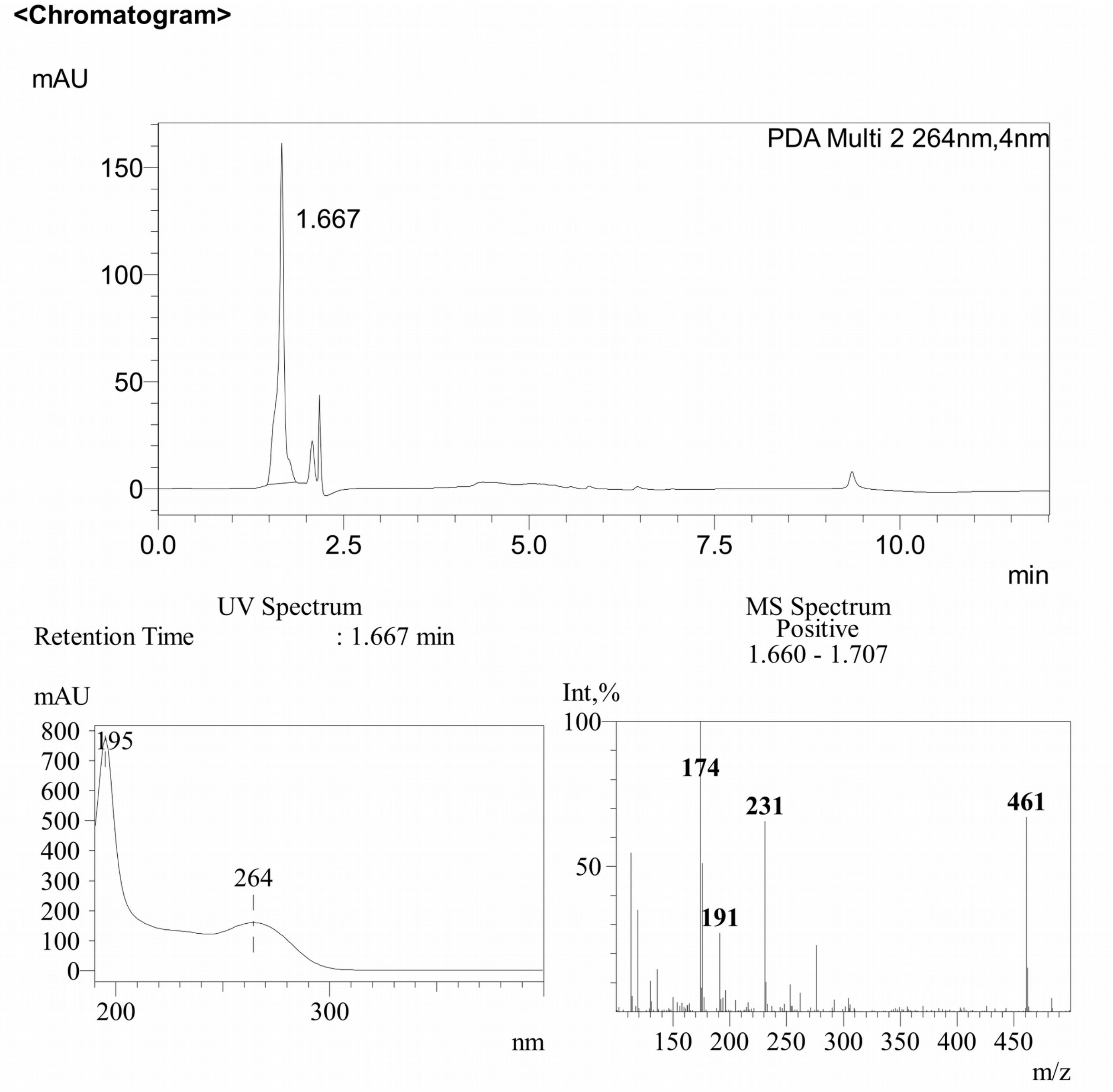

Figure S7. HPLC-MS analysis of the semicarbazide derivatized 34DHP bioconvesion product. The bioconversion was carried out with cell-free extracts of *E. coli* BL21(DE3) harboring pET-*kpiC* and pET-*kpiB* plasmids at 30 °C for 20 minutes, and then the reaction mixture was derivatized with semicarbazide. m/z 174 $[M+H]^+$ represents a 3-formylpyruvate derivatized with one molecule of semicarbazide, m/z 191 $[M+NH_4]^+$ is an NH_4^+ adduct of 174, m/z 231 $[M+H]^+$ represents a 3-formylpyruvate derivatized with two molecules of semicarbazide, m/z 461 $[2M+H]^+$ is a dimer of 231.

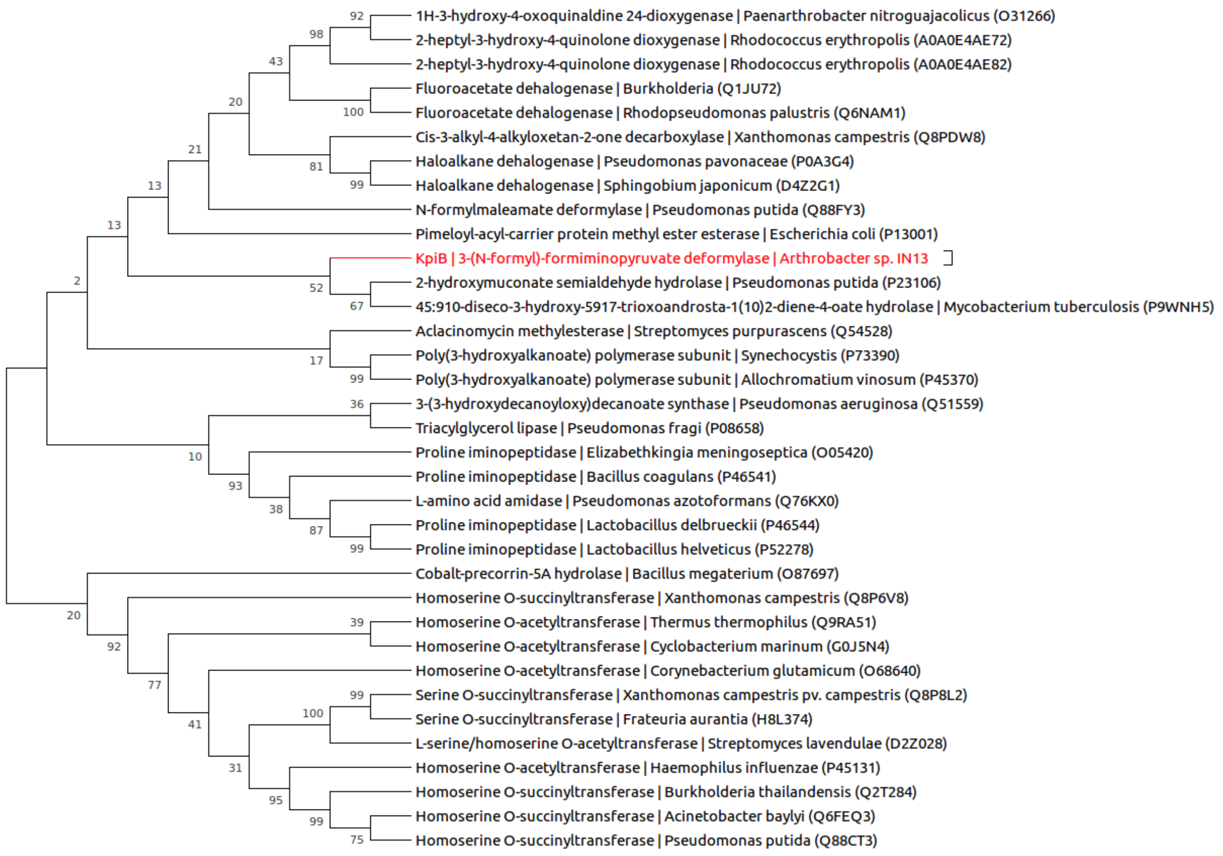

Figure S8. The phylogenetic analysis for KpiB in comparison with selected enzymes from α/β hydrolase fold superfamily (Pfam00561). Proteins were selected from UniProtKB database using determinants: pf00561 and bacteria and annotation:(type:"catalytic activity" evidence: "Inferred from experiment") and reviewed:yes. The evolutionary history was inferred by using the Maximum Likelihood method and JTT matrix-based model [4]. The bootstrap consensus tree inferred from 300 replicates [5] is taken to represent the evolutionary history of the taxa analyzed [5]. Branches corresponding to partitions reproduced in less than 50% bootstrap replicates are collapsed. The percentage of replicate trees in which the associated taxa clustered together in the bootstrap test (300 replicates) are shown next to the branches [5]. Initial tree(s) for the heuristic search was obtained automatically by applying Neighbor-Join and BioNJ algorithms to a matrix of pairwise distances estimated using the JTT model, and then selecting the topology with superior log likelihood value. This analysis involved 35 amino acid sequences. Evolutionary analyses were conducted in MEGA X [6].

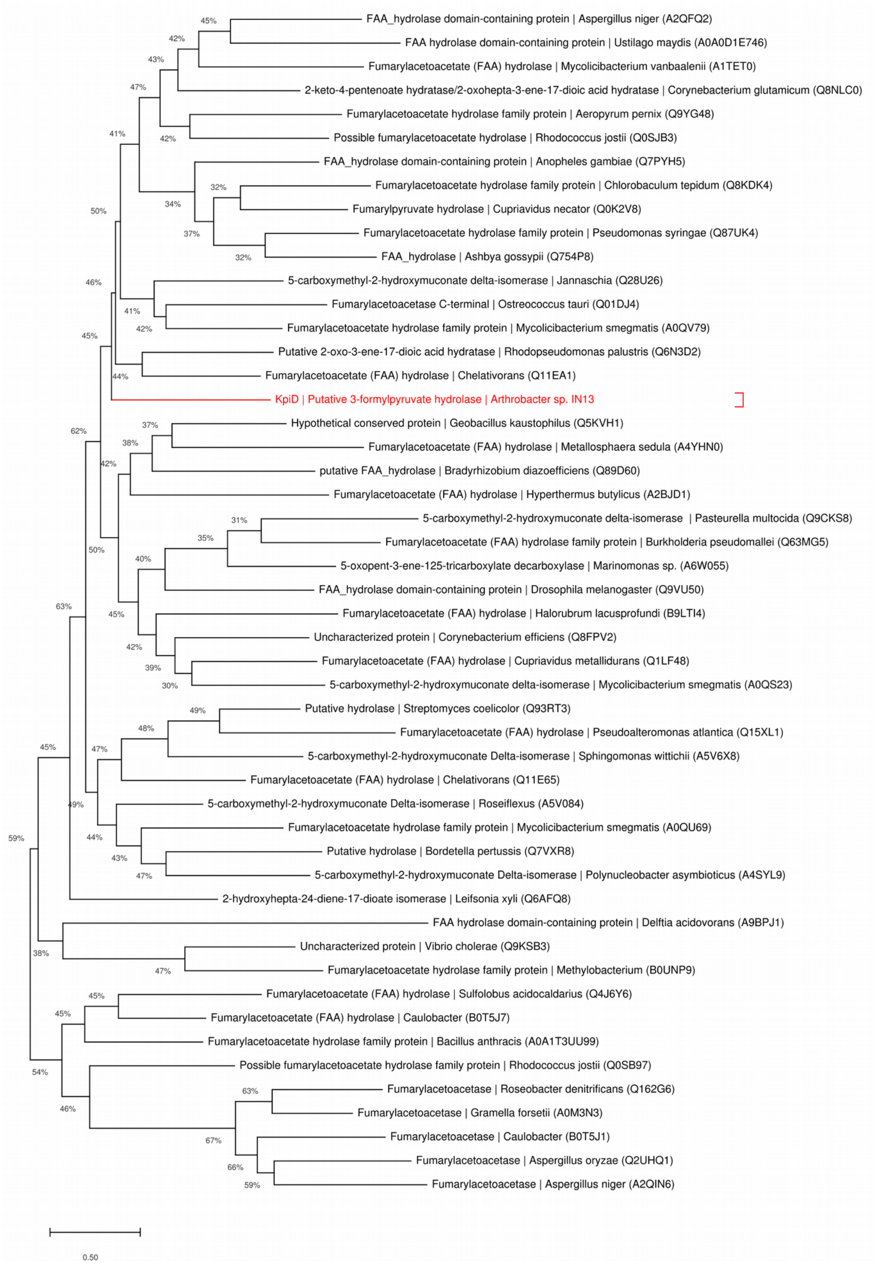

Figure S9. The phylogenetic analysis for KpiD in comparison with selected enzymes from fumarylacetoacetate hydrolase family (pfam01557). Proteins were selected from Pfam database, the provided seed alignment for Pfam01557 family. The evolutionary history was inferred by using the Maximum Likelihood method and JTT matrix-based model [4]. The tree with the highest log likelihood (-34803.58) is shown. The percentage of trees in which the associated taxa clustered together is shown next to the branches. Initial tree(s) for the heuristic search was obtained automatically by applying Neighbor-Join and BioNJ algorithms to a matrix of pairwise distances estimated using the JTT model, and then selecting the topology with superior log likelihood value. The tree is drawn to scale, with branch lengths measured in the number of substitutions per site. The proportion of sites where at least 1 unambiguous base is present in at least 1 sequence for each descendent clade is shown next to each internal node in the tree. This analysis involved 50 amino acid sequences. Evolutionary analyses were conducted in MEGA X [6].

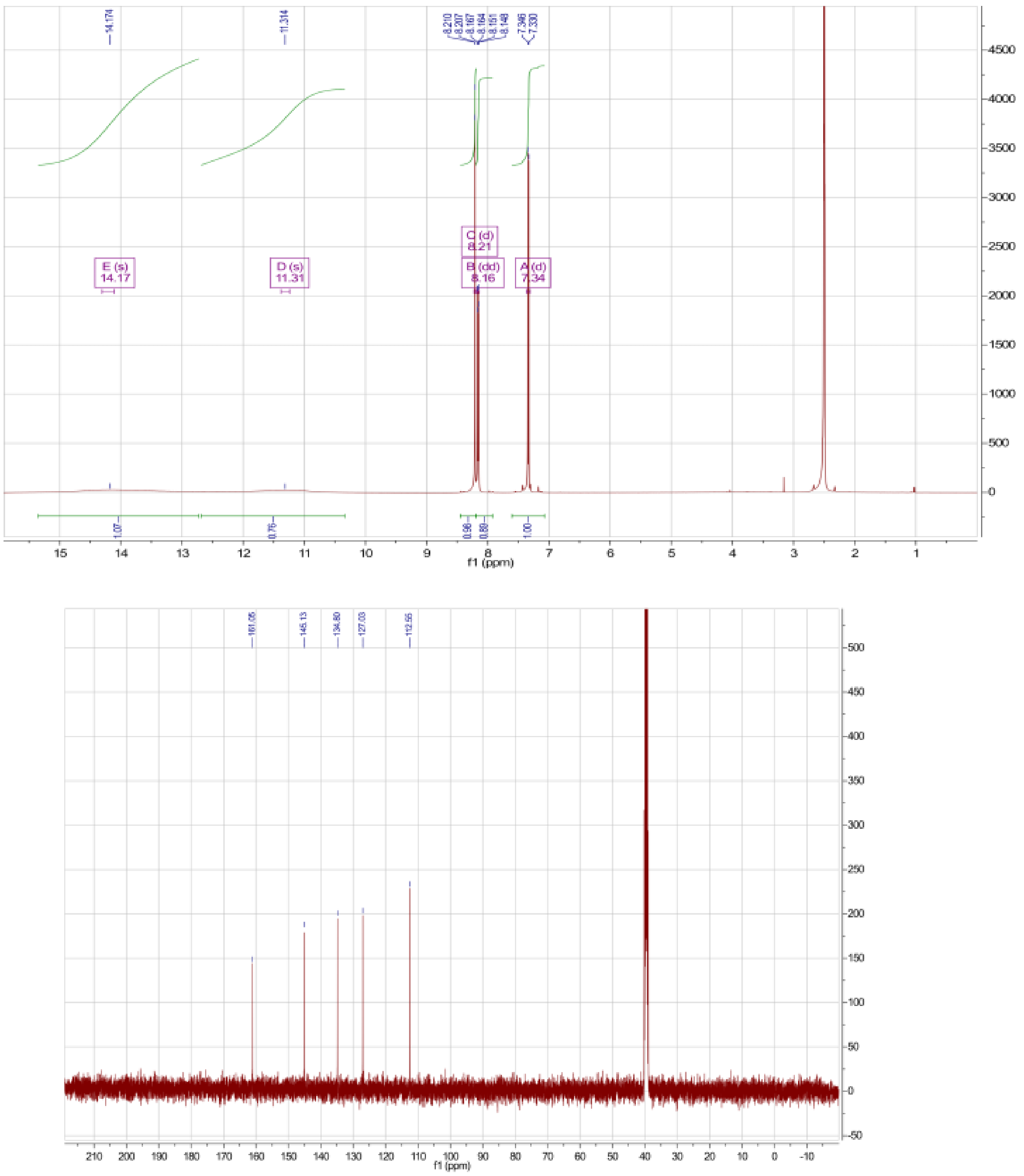

Figure S10. ^{1}H and ^{13}C NMR spectra of chemically synthesized pyridine-3,4-diol hydrochloride.

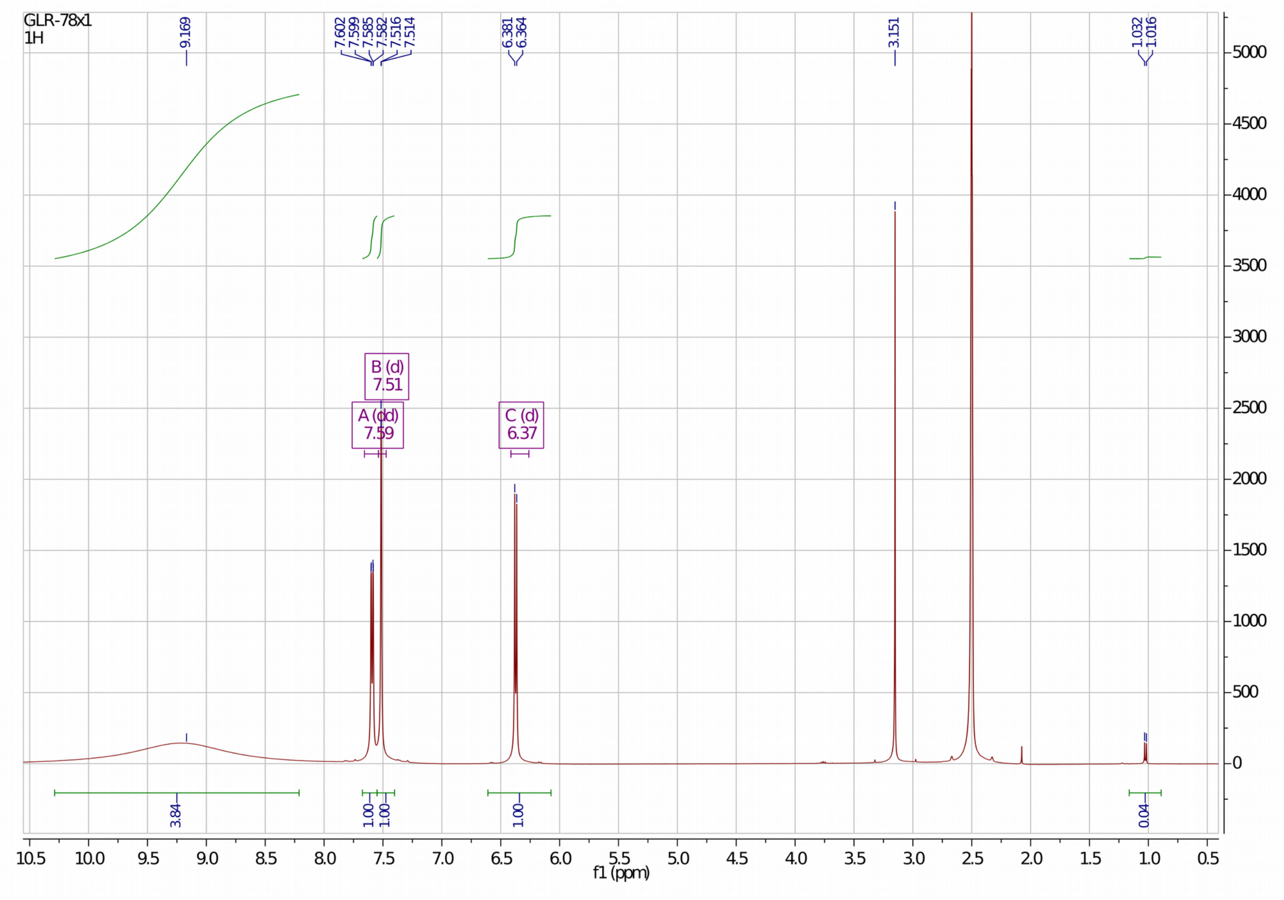

Figure S11. ^{1}H NMR spectra of chemically synthesized 3,4-dihydroxypyridine.

Supplementary references

1. Nakashima, N.; Tamura, T. Isolation and characterization of a rolling-circle-type plasmid from *Rhodococcus erythropolis* and application of the plasmid to multiple-recombinant-protein expression. *Appl. Environ. Microbiol.* **2004**, *70*, 5557–68, doi:10.1128/AEM.70.9.5557-5568.2004.

2. Gasparavičiūtė, R.; Kropa, A.; Meskys, R. A new *Arthrobacter* strain utilizing 4-hydroxypyridine. *Biologija* **2006**, 41–45.

3. Quan, S.; Dabbs, E.R. Nocardioform arsenic resistance plasmid characterization and improved *Rhodococcus* cloning vectors. *Plasmid* **1993**, *29*, 74–9, doi:10.1006/plas.1993.1010.

4. Jones, D.T.; Taylor, W.R.; Thornton, J.M. The rapid generation of mutation data matrices. *Comput Appl Biosci.* **1992**, doi:doi.org/10.1093/bioinformatics/8.3.275.

5. Felsenstein, J. Confidence Limits on Phylogenies: An Approach Using the Bootstrap. *Evolution (N. Y).* **1985**, 783–791, doi:10.2307/2408678.

6. Kumar, S.; Stecher, G.; Li, M.; Knyaz, C.; Tamura, K. MEGA X: Molecular evolutionary genetics analysis across computing platforms. *Mol. Biol. Evol.* **2018**, *35*, 1547–1549, doi:10.1093/molbev/msy096.

7. Saitou, N.; Nei, M. The neighbor-joining method: a new method for reconstructing phylogenetic trees. *Mol. Biol. Evol.* **1987**, *4*, doi:10.1093/oxfordjournals.molbev.a040454.

8. Zuckerkandl, E.; Pauling, L. Evolutionary Divergence and Convergence in Proteins. In *Evolving Genes and Proteins*; **1965**.
